# Survey-based evidence on occupational fraud exposure, reporting behavior, and informal norms in Romania, Ukraine, and the Republic of Moldova

**DOI:** 10.3389/fpubh.2026.1923314

**Published:** 2026-09-25

**Authors:** Lucia Morosan-Danila, Daniela-Mihaela Neamtu, Claudia-Elena Grigoras-Ichim, Denisa-Oana Zelinschi

**Affiliations:** 1 Department of Accounting, Auditing and Finance, Faculty of Economics, Administration and Business, “Stefan cel Mare” University of Suceava, Suceava, Romania; 2 Department of Management, Business Administration and Tourism, Faculty of Economics, Administration and Business, “Stefan cel Mare” University of Suceava, Suceava, Romania; 3 Department of Maternal and Child Medicine, Faculty of Medicine, “Grigore T. Popa” University of Medicine and Pharmacy, Iasi, Romania

**Keywords:** cross-national survey, Eastern Europe, fraud perception, informal workplace norms and corruption tolerance, internal control, multiple correspondence analysis, occupational fraud, whistleblowing

## Abstract

**Background:**

Occupational fraud is usually examined as a financial control and governance problem, but it may also be relevant to occupational and organizational health through reporting culture, perceived accountability, and workplace governance. However, this study does not measure health outcomes directly.

**Methods:**

A cross-sectional online survey (19 items) was administered between September 2025 and February 2026 to 1,007 employed adults (Romania = 407; Ukraine = 350; Moldova = 250). Differences among surveyed national subsamples were tested with *χ*^2^ tests (nominal items) and Kruskal-Wallis tests (ordinal items) with Holm adjustment. Covariate-adjusted ordinal and binary logistic regression models were estimated to assess whether selected fraud-related outcomes remained associated with national subsample membership after adjustment for age, gender, residence environment, organization type, and organization size. Multiple Correspondence Analysis, followed by k-means clustering, was used to explore aggregated response typologies.

**Results:**

Perceived fraud typology differed significantly across surveyed national subsamples (*χ*^2^ = 58.16, *p* < 0.001; Cramér’s *V* = 0.17). Among surveyed respondents from Ukraine, higher-frequency proposals to engage in potentially fraudulent or unethical activities were more common (ITM_4, categories 3–5: 28.6% vs. 18.2% in Romania and 17.2% in Moldova). Personal victimization was also highest among surveyed respondents from Ukraine (14.6%), whereas indirect victimization, measured as knowing victimized colleagues, was highest among respondents from Moldova (46.4%). A two-cluster solution separated a large “lower-exposure/refusal-dominant” profile from a smaller “higher-exposure/non-refusal-enriched” profile that was more frequent among respondents from Ukraine and Moldova. In adjusted outcome models, several contrasts persisted: respondents from Ukraine showed higher odds of fraud-related/unethical proposal frequency, perceived workplace gift/hospitality acceptance, and selecting no action rather than notifying management (OR = 3.82, 95% CI 1.65–8.85), whereas respondents from the Republic of Moldova showed higher odds of personal/indirect victimization and perceived colleague involvement.

**Conclusion:**

Among surveyed respondents, exposure, reporting behavior, and informal workplace norms varied across the three national subsamples. The findings cautiously suggest that anti-fraud interventions may benefit from considering the reporting cultures and perceived exposure patterns observed in each surveyed subsample. However, because the sample was non-probabilistic and effect sizes were generally small, policy implications should be interpreted as exploratory. They should not be generalized to national populations without further representative research.

## 1 Introduction

Occupational fraud is one of the most persistent challenges of contemporary organizational governance, affecting both the public and private sectors. Occupational fraud is commonly defined as the use of a professional position to obtain personal benefit through the misappropriation or misuse of organizational resources (1). The literature delineates three main forms: asset misappropriation, corruption, and financial reporting fraud. Although asset misappropriation is the most common, financial statement fraud generates the highest average losses (1). The dominant conceptual foundation is the “Fraud Triangle” theory, which posits that fraud occurs when pressure, opportunity, and rationalization coexist (2). The model emphasizes the role of opportunity arising from deficiencies in internal control, an element considered most susceptible to managerial intervention (3). The extension of this model through the “Fraud Diamond” introduces the capability dimension, arguing that the perpetrator must have the position and skills necessary to exploit the system (4). Weaknesses in segregation of duties, poor monitoring, and the absence of internal audit significantly increase the risk of fraud (5). Deficiencies in board oversight and the audit function are also frequently associated with major financial fraud (6–8). At the same time, perceived ethical norms and leadership behavior influence the likelihood of unethical behavior (9). A strong ethical climate reduces fraudulent behavior, while the perception that such practices are tolerated increases the risk of deviance (10). The process of “normalization of corruption” explains how initially deviant behaviors can become informally accepted in organizations (11). More recent approaches include factors such as arrogance or a sense of superiority, suggesting a more complex behavioral profile of the fraudster (12). This standard occupational-fraud typology provides the conceptual basis for interpreting the fraud-related items included in the questionnaire (1, 13–19).

Beyond direct financial losses, it undermines institutional trust, erodes ethical culture, and generates reputational costs that are difficult to quantify. In an economic environment marked by accelerated technological transformations, competitive pressures, and geopolitical volatility, organizational vulnerabilities become more complex, and traditional control mechanisms are often tested. Thus, the analysis of occupational fraud can no longer be limited to the accounting or legal dimension, but requires an interdisciplinary approach that integrates organizational, behavioral, and institutional perspectives. Bibliometric research has mapped economic fraud and its associated risks (40), while complementary work combines the mapping of financial-fraud research with an analysis of the Romanian and EU legislative context (42).

In Eastern Europe, the issue of occupational fraud is particularly relevant. In recent decades, the region has undergone profound economic transitions, institutional reforms, and geopolitical realignments, which have influenced organizational structures, managerial practices, and compliance culture. Differences in institutional consolidation, the level of digitalization, the maturity of internal control systems, and the degree of social trust can generate significant variations in how fraud is perceived, reported, and managed. At the same time, progressive integration into European and international economic circuits has imposed higher standards of transparency and accountability, creating a tension between the formalization of procedures and their practical efficiency. This context supports an exploratory comparison of nationally surveyed subsamples from Eastern Europe, focusing on perceived fraud-related patterns and organizational contexts.

Therefore, to move from a general regional description to a contextualized analysis, we briefly present the main features influencing the risk of fraud in each of the three countries analyzed. In Romania, EU membership has contributed to a more formalized compliance and reporting framework, including the transposition of EU sustainability reporting requirements under the CSRD (20, 21). The expansion of digital public-service platforms, including the national online payment platform Ghișeul.ro, also increases the traceability of selected interactions with public institutions. However, persistent vulnerabilities in the informal economy and uneven implementation capacity continue to limit the effectiveness of formal compliance mechanisms (22).

Anti-corruption reforms and institutional independence remain central issues in the context of Ukraine’s governance and EU accession (23). However, the government continues to promote the digitalization agenda; the Diia platform and the Integrity Strategy 2023–25 include 63 digital tools for fraud prevention (22). This tension between pro-integrity reforms and geopolitical constraints creates a still volatile internal control framework.

In the Republic of Moldova, the path of European convergence is reflected in the adoption of the Public Internal Financial Control (PIFC) framework, which the European Commission has positively assessed. However, administrative capacity remains limited (24). According to the U.S. Investment Climate Report, the informal economy is estimated at around 23% of GDP, particularly in agriculture and construction, undermining the effectiveness of formal compliance mechanisms (25). Consequently, strengthening internal audit and expanding electronic payments are key priorities to reduce opportunities for fraud.

Although occupational fraud is primarily a governance and financial-control issue, its reporting and organizational dimensions may also be relevant to occupational health and safety. Reporting intentions, informal workplace norms, and perceived organizational responses can provide indirect information about the governance environment in which employees operate. The present study does not directly measure psychological safety, retaliation, occupational stress, or health outcomes; therefore, this framing is organizational rather than clinical or health-outcome based. Theoretically and practically, occupational fraud is closely linked to the dynamics of organizational opportunities, individual pressures, and informal norms that shape employee behavior. In many organizations, the formal existence of anti-fraud procedures coexists with different perceptions of their effectiveness. For example, small organizations are vulnerable due to insufficient separation of duties, whereas large organizations can facilitate the concealment of complex fraud through operational layering (1). Additionally, direct or indirect exposure to fraudulent practices can influence employees’ attitudes, reporting behavior, and tolerance for deviant behavior. Across different national contexts, these variables can interact in distinct ways, generating specific patterns of vulnerability and organizational responses.

Conceptually, the study uses the Fraud Triangle and Fraud Diamond as orienting frameworks rather than as fully operationalized causal models. The opportunity is approached through items related to perceived financial misstatements, anti-fraud procedures, reporting mechanisms, and fraud risk management. Rationalization and informal norms are approached through responses to superior-requested fraud and perceived colleague involvement. Organizational context is captured by organization type (private/public sector), organization size, and reporting channel preferences. Because pressure and individual capability were not directly measured, these frameworks guide the interpretation of response patterns rather than serving as validated latent constructs.

Within this framework, this paper investigates transnational differences in the experiential and organizational dimensions of occupational fraud, with a focus on Eastern European countries. The approach is structured in four main parts. The first part introduces the general theme and its relevance in the regional and organizational context. The second part presents the research design, including the methodology used, the statistical analysis tools, and the justification of the methodological choices. The third part presents the empirical results, focusing on cross-country differences and the identified typologies. Finally, the fourth part discusses the theoretical and practical implications of the findings, formulates the conclusions, and highlights future research directions.

Despite the growing literature on occupational fraud and internal control, comparative micro-level evidence from Eastern Europe remains limited. Existing studies tend to focus either on single-country contexts or on broad corruption indicators, without simultaneously examining exposure, reporting behavior, fraud typologies, and informal workplace norms. This study addresses this gap by comparing Romania, Ukraine, and the Republic of Moldova using survey data from 1,007 respondents. This study contributes by:

- Providing recent comparative microdata on occupational fraud perceptions in three Eastern European contexts;
- Identifying sample-level differences among surveyed respondent groups in exposure, victimization, reporting, and perceived gift/hospitality acceptance practices;
- Exploring aggregate response profiles using MCA and clustering.

The purpose of the research is the comparative assessment of occupational fraud (perceptions, exposure, norms/acceptability, and organizational mechanisms for prevention/ detection/ reporting) in Romania, Ukraine, and the Republic of Moldova, as well as the identification of factors that statistically differentiate the three surveyed national subsamples, including through covariate-adjusted exploratory comparisons and aggregate response typologies. In this context, the objectives of the research are:

*O1*: To describe the level and forms of occupational fraud reported/perceived in the total sample and in each surveyed national subsample (Romania, Ukraine, and the Republic of Moldova), through descriptive indicators (frequencies/percentages) and comparative distributions.

*O2*: To test the existence of significant differences among the three surveyed national subsamples regarding exposure/perceptions and acceptability-related response patterns towards occupational fraud, using inferential tests appropriate to the type of variable (χ^2^ for categorical items and Kruskal-Wallis for ordinal items), with reporting of effect size and control of type I error under multiple testing conditions.

*O3*: To comparatively analyze reporting intentions, preferred fraud-risk management responses, perceived informal workplace practices, and the reported presence of anti-fraud procedures, highlighting the dimensions with practical relevance for anti-fraud governance.

*O4*: To assess whether selected differences in fraud-related outcomes across surveyed national subsamples persist after adjustment for respondent and organizational characteristics, using ordinal and binary logistic regression models, and to explore aggregate response typologies using MCA followed by clustering.

The research hypotheses resulting from the analysis of the literature and the specific methodology are broken down:

*H1*: *The distribution of responses regarding exposure to and perception of occupational fraud differs significantly across the three surveyed national subsamples, particularly on items capturing encounters with misrepresentations/violations, the frequency of proposals to engage in potentially fraudulent or unethical activities, and the experience of victimization*. Recent literature indicates that exposure to and perception of fraud vary across countries, depending on the effectiveness of internal controls and organizational culture, which generate different levels of opportunity and tolerance for fraudulent behavior (26–30).

*H2*: *Attitudes and acceptability toward practices associated with occupational fraud differ significantly across the three surveyed national subsamples, as reflected in forced-choice responses to a superior’s request to commit fraud and in perceptions of collective-level informal norms*. These patterns may be interpreted in the context of prior literature on ethical climate, informal norms, and perceived sanctioning environments, which may influence acceptability-related response patterns and the tendency to justify violations (26, 28–30).

*H3: Reporting intentions, preferred fraud-risk management responses, and perceived gift/hospitality practices differ across the three surveyed national subsamples.* These differences may reflect the maturity of corporate governance and compliance practices, respectively, and institutional variations that shape both the willingness to report and the perception of the costs associated with fraud (26–31).

*H4: After adjustment for respondent and organizational characteristics, national subsample membership remains associated with selected fraud-related outcomes, including fraud-related/unethical proposal frequency, victimization, reporting responses, conditional acceptance/refusal responses to superior-requested fraud, perceived colleague involvement, and perceived workplace gift/hospitality acceptance.* This hypothesis is interpreted as an exploratory adjusted association, not as causal evidence or as nationally representative country-level inference. The hypothesis is grounded in prior research linking occupational fraud to opportunity structures, internal control environments, ethical climate, reporting behavior, and informal organizational norms (6, 8, 26–29, 32).

## 2 Materials and methods

### 2.1 Research design

The study adopts a quantitative, cross-sectional design to capture a snapshot of occupational fraud experiences across three neighboring Eastern European countries. Data were collected online between September 2025 and February 2026 using a 19-item self-administered questionnaire (8 socio-demographic/organizational items and 11 thematic items; ITM 1–11). A convenience-snowball strategy was chosen because no sampling frame covering employees across all three surveyed subsamples exists; invitations were circulated via university mailing lists and professional social media groups, with respondents encouraged to forward the link. Eligibility criteria required participants to be ≥18 years old, to have been employed full- or part-time in the previous 12 months, and to report that their company/institution was based in Romania, Ukraine, or the Republic of Moldova. National subsample membership was operationalized according to the country of the respondent’s employing organization. A 24-person pilot test (8 per country) checked clarity, timing, and intercultural equivalence of the translations; minor lexical adjustments were made before launch. The final analytic sample comprised 1,007 valid cases (Romania = 407; Ukraine = 350; Moldova = 250) after excluding submissions with >40% missing data. Full details on sampling, instrument structure, and exclusions are provided in Sections 2.2–2.3.

### 2.2 Participants and sampling

We employed a non-probability convenience-snowball design. The survey link was initially distributed via the official mailing lists of the universities involved in each country (e.g., “Stefan cel Mare” University of Suceava) and reposted in social groups dedicated to accounting, finance, and management professionals. Participants were encouraged to forward the invitation to colleagues, generating a snowball recruitment.

Eligible respondents had to (i) be ≥18 years old, (ii) have been employed full- or part-time in the last 12 months, and (iii) report that the company/institution in which they worked was based in Romania, Ukraine, or the Republic of Moldova. National subsample membership was assigned using the reported country of the company/institution, not citizenship or personal country of residence. Students with no work experience and retirees were excluded at the screening stage.

Although Google Forms may generate technical metadata, the research team did not export, store, analyze, or link IP addresses to survey responses. Responses with >40% missing items (*n* = 11) were removed. Data were collected using a standardized questionnaire, and the instrument was available in the participants’ national languages. A pilot study with 24 participants (8 per country) assessed clarity and timing; minor wording adjustments were made (e.g., simplified legal terms). Pilot data were not included in the final analyses. The questionnaire was administered in Romanian in Romania and the Republic of Moldova and in Ukrainian in Ukraine. Translation and language adaptation were reviewed by bilingual members/collaborators of the research team familiar with accounting and fraud terminology. Cross-linguistic adaptation was addressed through language-specific versions of the questionnaire, review of item wording by the research team, and pilot testing in each national subsample. The pilot assessed clarity, timing, professional terminology, and the comparability of item meaning across language versions. Minor wording adjustments were made before data collection. However, no formal back-translation procedure or statistical measurement-invariance testing was performed; therefore, measurement equivalence should be understood as face/content equivalence supported by pilot feedback, not as formal psychometric equivalence.

A total of 1,018 submissions were received; after applying the exclusion rules above, the analytic sample comprised 1,007 valid cases (Romania = 407; Ukraine = 350; Moldova = 250). The sample comprises respondents from diverse organizational environments, and the variables analyzed encompass both socio-demographic and organizational characteristics, as well as items on exposure, perceptions, acceptability, reporting, and organizational mechanisms associated with occupational fraud.

Because the strategy was non-probabilistic, representativeness cannot be guaranteed; the external validity and generalizability of the findings are therefore limited to similar respondent profiles and organizational contexts. Findings should be interpreted as exploratory and hypothesis-generating rather than as nationally representative estimates.

### 2.3 Questionnaire and measures

The questionnaire was designed to capture item-level indicators of occupational fraud perception, exposure, reporting intentions, informal workplace norms, and acceptability-related response patterns. It was not intended to operate as a validated multi-item psychometric scale. The instrument included 19 items: 8 socio-demographic and organizational items and 11 thematic items related to occupational fraud (ITM_1–ITM_11). Most thematic variables were measured through single items, using nominal/dichotomous, multiple-choice, or ordinal 1–5 response formats. Two items required cautious interpretation because their wording and response format were not fully symmetrical. ITM_1 was analyzed only as a nominal perceived-association item, not as a graded measure of the degree to which occupational fraud and corruption are related. ITM_7 was analyzed as a forced-choice nominal item distinguishing refusal, situational dependence, and conditional acceptance with personal benefit; it was not interpreted as a continuous probability of compliance or as a separate measure of profit motive. The pilot study assessed clarity, timing, and intercultural comprehensibility, but did not constitute formal psychometric validation. Because the questionnaire was developed for this exploratory survey and did not use previously validated multi-item psychometric scales, validity and reliability were addressed in a limited, instrument-specific manner. Face validity and content relevance were assessed through the pilot test and by aligning item content with occupational-fraud concepts discussed in the literature, including exposure, perceived norms, reporting intentions, and organizational prevention mechanisms. Reliability coefficients such as Cronbach’s alpha were not calculated because the thematic items were not intended to form a unidimensional scale or composite index; instead, they were analyzed as separate item-level indicators using response formats appropriate to each item. The questionnaire was completed voluntarily by *N* = 1,007 respondents from Romania (*n* = 407), Ukraine (*n* = 350), and the Republic of Moldova (*n* = 250), representing public and private organizations across various functions and fields of activity. Because several thematic items are repeatedly referenced in the Results and Discussion by code, Table 1 summarizes the key items used in the main analyses. The full item-level measurement overview is provided in Supplementary Table 1.

**Table 1:** Key thematic items referenced in the main analyses.

Code	Short label	Full item	Response type
ITM_2	Financial distortions	At the company/institution where you work, have you encountered cases where significant misstatements or other violations of financial statements have been committed?	Yes / No / Do not know or no answer
ITM_4	Fraud-related/unethical proposal frequency	In the last 3 years, how often have you been asked to participate in activities that could be considered fraudulent or unethical?	Ordinal 1–5 scale
ITM_5	Victimization	Have you been the victim of fraud committed by someone you trusted?	Never / No, but I know colleagues who were / Yes
ITM_6	Reporting channel	If you became aware of occupational fraud in your organization, whom would you contact first?	Manager / OLAF or Fiscal State Service / Nothing
ITM_7	Conditional acceptance/refusal	If a superior asked you to participate in a fraudulent act, how likely would you be to comply?	No / Depends on the situation / Yes, if I also benefit
ITM_8	Colleague practices	Have you observed colleagues engaging in practices that may be considered fraudulent or unethical?	Yes / No / Do not know or no answer
ITM_9	Fraud typology	What are the most common types of fraud?	Multiple nominal categories
ITM_11	Gift/hospitality acceptance frequency	How often are gifts or hospitality from clients accepted in your workplace?	Ordinal 1–5 scale

### 2.4 Data processing

The statistical analysis was performed in Python (v.3.10), using pandas 2.2, scipy 1.13, statsmodels, scikit-learn 1.4, pingouin 0.5.4, and prince-mca 0.7, based on the database exported from Google Forms and used in .xlsx format (*N* = 1,007), after homogenizing the labels, especially standardizing the organizational-country variable used to assign national subsample membership and checking the consistency of the codings, while keeping all observations. More specifically, of the total 1,018 questionnaires received, 11 were eliminated because they contained more than 40% unanswered questions. For the ordinal 1–5 items, missingness was minimal, with no item exceeding 0.3% missing values. These values were imputed using the country-specific median. Sensitivity analyses using complete-case data and Random Forest imputation led to unchanged substantive conclusions. For categorical items, the non-response options “Prefer not to answer”, “I do not know” and “Not applicable” were kept as distinct options; the residual technical missingness was coded as such and did not influence the descriptive analyses.

Full questionnaire coding, full covariate-adjusted model estimates, proportional-odds diagnostics, detailed Likert distributions, and cluster-profile contrasts are provided in Supplementary Tables 1−5.

### 2.5 Statistical analysis and robustness checks

Differences among surveyed national subsamples for nominal and dichotomous thematic items were assessed using *χ*^2^ tests of independence, based on National subsample × Response contingency tables. For completeness, *χ*^2^ tests were also used to compare the full response-category distributions of the two ordinal 1–5 items (ITM_4 and ITM_11), and these distributional *χ*^2^ results are reported in Table 2. However, because these items have an ordered response structure, Kruskal-Wallis tests were treated as the primary ordinal analyses. This approach was used for item-level comparisons linked to H1–H3 and followed the frequency-comparison logic commonly used in occupational-fraud reporting. Cramér’s *V* was reported as the effect-size measure, and Holm-adjusted *p*-values were used to control for multiple comparisons (33). To avoid inflation caused by low expected counts, response categories occurring in ≤3% of the total sample were flagged as rare cells. For ITM_9, which originally included 14 response categories, a sensitivity analysis aggregated the three most frequent categories and grouped all remaining responses into an “Other/rare” category, yielding a four-category version, in line with robustness concerns for sparse categorical data (34). The *χ*^2^ test was then repeated for this aggregated version using both asymptotic and Monte Carlo *p*-values.

**Table 2:** *χ*^2^ tests by national subsample.

Item	*N*	No of categories	*χ* ^2^	df	*p*	p (Holm)	Cramér *V*
ITM 5	1,007	3	32.553	4	0.000001	0.000016	0.127
ITM 2	1,007	3	31.147	4	0.000003	0.000029	0.124
ITM 4	1,007	5	39.142	8	0.000005	0.000042	0.139
ITM 9	1,007	14	58.158	26	0.000295	0.002361	0.170
ITM 11	1,007	5	25.880	8	0.001101	0.007708	0.113
ITM 6	1,007	3	16.722	4	0.002189	0.013134	0.091
ITM 8	1,007	3	14.790	4	0.005158	0.025788	0.086
ITM 7	1,007	3	14.215	4	0.006640	0.026561	0.084
ITM 10	1,007	4	14.763	6	0.022184	0.066551	0.086
ITM 1	1,007	3	6.428	4	0.169400	0.338800	0.056
ITM 3	1,007	3	2.962	4	0.564121	0.564121	0.038

^*^p(Holm) represents the *p*-value adjusted by Holm’s correction for multiple testing; Cramér’s V indicates the effect size.

For the two Likert-type items (ITM 4 and ITM 11), differences among surveyed national subsamples were tested with the Kruskal-Wallis rank-sum test. When the global test was significant, pairwise contrasts were examined using Mann–Whitney tests with Holm adjustment. Effect magnitude was expressed as *ε*^2^ (computed with scipy 1.13). Frequencies of exposure to unethical behavior were operationalized according to Kaptein (10) and interpreted in light of the hierarchical comparisons proposed by Treviño et al. (9). Detailed distributions by response categories are shown in Supplementary Table 3.

To address the possibility that crude national subsample comparisons were confounded by differences in respondent composition, additional covariate-adjusted outcome models were estimated (35). In these models, selected fraud-related survey items were treated as dependent variables, while national subsample membership was included as a predictor, with Romania as the reference category. Models were adjusted for age group, gender, residence environment, organization type (private/public sector), and organization size. Covariates were selected *a priori* because they differed across national subsamples and could plausibly confound associations between national subsample membership and fraud-related responses.

Ordinal logistic regression was used for the two 1–5 frequency items: ITM_4, measuring the frequency of proposals to engage in potentially fraudulent or unethical activities, and ITM_11, measuring the perceived frequency of acceptance of workplace gifts/hospitality. For these ordinal models, the proportional-odds/parallel-lines assumption was assessed using threshold-specific cumulative binary logistic regressions fitted at each cut-point with the same covariates. Because the diagnostics focused on the stability of the national-subsample contrasts, multinomial logistic regression models treating ITM_4 and ITM_11 as nominal outcomes were also estimated as sensitivity analyses. The ordinal models were retained as the main parsimonious specification, and the sensitivity analyses were used to assess whether the substantive interpretation was robust to relaxing the proportional-odds assumption. Binary logistic regression was used for prespecified contrasts derived from ITM_5, ITM_6, ITM_7, and ITM_8. For ITM_5, personal victimization was contrasted with never being victimized, and indirect victimization was contrasted with never being victimized. Because ITM_5 includes three substantive response categories, a supplementary multinomial logistic regression model retaining all categories (never, indirect victimization, and personal victimization) was also estimated as a sensitivity analysis using the same covariates. For ITM_6, external/formal reporting and no action were each contrasted with notifying management. Because ITM_5, ITM_6, and ITM_7 each include three substantive nominal response categories, supplementary penalized multinomial logistic regression models that retain all categories were also estimated as sensitivity analyses, using the same covariates. For ITM_6, the supplementary multinomial model used an L2 penalty because the no-action category was sparse and the corresponding unpenalized model produced a singular matrix. For ITM_7, the binary contrast compared any non-refusal response (“Depends on the situation” or “Yes, if I also benefit”) with refusal (“No”). This contrast was interpreted as a forced-choice conditional-acceptance pattern, not as a continuous probability of compliance or as a separate measure of profit motive. For ITM_8, perceived colleague involvement was contrasted with no involvement or non-affirmative responses. As a sensitivity check, the ITM_8 model was re-estimated after excluding uncertain or non-response categories and contrasting affirmative responses only with explicit “No” responses.

Respondents aged over 60 were grouped with the 46–60 category because of sparse counts, and observations with non-informative gender or organization-type responses were excluded from the adjusted models. Categorical predictors in the adjusted models were dummy-coded using treatment coding with one omitted reference category. The reference categories were Romania for national subsample membership, age under 25 years, female gender, rural residence, private-sector organization, and organizations with fewer than 10 employees. Reported odds ratios for non-reference categories are therefore interpreted relative to these omitted categories. Odds ratios, 95% confidence intervals, and Holm-adjusted *p*-values for national-subsample contrasts were reported. For the adjusted outcome analyses, Holm correction was applied across the 16 prespecified national-subsample contrasts reported in Table 3, corresponding to two national-subsample contrasts (Ukraine vs. Romania and Republic of Moldova vs. Romania) across eight modeled outcome contrasts. These models were interpreted as exploratory adjusted associations, not as causal or nationally representative estimates.

**Table 3:** Covariate-adjusted national-subsample contrasts for selected fraud-related outcomes.

Outcome	Model	Level_or_event	*N*	Contrast	OR	CI_low	CI_high	*p*	p_Holm_country_contrasts
ITM4_fraud_related_unethical_proposal_frequency	Ordinal logistic regression	Higher fraud-related/unethical proposal frequency	987	Ukraine vs. Romania	2.277499	1.630594	3.18105	1.38E-06	2.07E-05
ITM4_fraud_related_unethical_proposal_frequency	Ordinal logistic regression	Higher fraud-related/unethical proposal frequency	987	Republic of Moldova vs. Romania	1.068002	0.757536	1.505708	0.707352	0.894223
ITM11_gift_hospitality_acceptance_frequency	Ordinal logistic regression	Higher perceived gift/hospitality acceptance frequency	987	Ukraine vs. Romania	1.76584	1.281882	2.43251	0.000502	0.00653
ITM11_gift_hospitality_acceptance_frequency	Ordinal logistic regression	Higher perceived gift/hospitality acceptance frequency	987	Republic of Moldova vs. Romania	1.320382	0.952403	1.830536	0.095437	0.34712
ITM5_personal_victimization	Binary logistic regression	Personal victimization vs. never	603	Ukraine vs. Romania	1.710235	1.016114	2.87852	0.043369	0.260213
ITM5_personal_victimization	Binary logistic regression	Personal victimization vs. never	603	Republic of Moldova vs. Romania	2.381488	1.363712	4.15886	0.002284	0.022845
ITM5_indirect_victimization	Binary logistic regression	Indirect victimization vs. never	862	Ukraine vs. Romania	1.642712	1.146534	2.353619	0.006825	0.054601
ITM5_indirect_victimization	Binary logistic regression	Indirect victimization vs. never	862	Republic of Moldova vs. Romania	2.514372	1.756531	3.599176	4.70E-07	7.52E-06
ITM6_external_vs_manager	Binary logistic regression	External/formal reporting vs. manager	944	Ukraine vs. Romania	1.420238	0.98051	2.057171	0.063476	0.317378
ITM6_external_vs_manager	Binary logistic regression	External/formal reporting vs. manager	944	Republic of Moldova vs. Romania	0.868793	0.604558	1.248519	0.447111	0.894223
ITM6_no_action_vs_manager	Binary logistic regression	No action vs. manager	668	Ukraine vs. Romania	3.822541	1.650205	8.85455	0.001756	0.021071
ITM6_no_action_vs_manager	Binary logistic regression	No action vs. manager	668	Republic of Moldova vs. Romania	0.310639	0.081505	1.18393	0.08678	0.34712
ITM7_non_refusal	Binary logistic regression	Non-refusal response vs. refusal	987	Ukraine vs. Romania	2.405591	1.379095	4.196136	0.001986	0.021849
ITM7_non_refusal	Binary logistic regression	Non-refusal response vs. refusal	987	Republic of Moldova vs. Romania	2.222468	1.291819	3.823574	0.003915	0.035236
ITM8_colleague_involvement_yes	Binary logistic regression	Colleague involvement yes vs. no/do not know	987	Ukraine vs. Romania	2.114872	1.184251	3.776804	0.011356	0.079493
ITM8_colleague_involvement_yes	Binary logistic regression	Colleague involvement yes vs. no/do not know	987	Republic of Moldova vs. Romania	2.819861	1.63164	4.873389	0.000204	0.002857

To identify aggregate response profiles, we applied Multiple Correspondence Analysis (MCA) to selected categorical items (ITM_2, ITM_5, ITM_6, ITM_7, ITM_8, and ITM_10), a dimensional-reduction method suitable for categorical data (36, 37). The resulting factorial coordinates were subjected to k-means clustering. Candidate cluster solutions were evaluated using silhouette and bootstrap stability diagnostics, while avoiding solutions driven by very small clusters. A two-cluster solution was retained. The association between cluster membership and national subsample membership was tested using *χ*^2^ and Cramér’s *V*.

## 3 Results

The statistical analyses described in the Methods section led to the following results. Interpretations of differences across surveyed national subsamples should be made with caution, because the respondent groups differ considerably in age and organizational profile; some observed variation may reflect cohort, recruitment channel, professional position, sector, or organizational composition rather than country-level effects.

In the analyzed sample (*N* = 1,007), according to Table 4, the distribution by country is as follows: Romania 40.4% (*n* = 407), Ukraine 34.8% (*n* = 350), and the Republic of Moldova 24.8% (*n* = 250). From a socio-demographic point of view, the age category under 25 years old predominates (53.2%), with high shares in Romania (69.0%) and the Republic of Moldova (68.0%). At the same time, in Ukraine, the structure is was older (24.3% under 25, and 36.9% are 46–60 years old). The sample is predominantly female (78.0%) and relatively uniform across countries (approximately 76–79%). As places of residence, rural areas are more common overall (57.4%), with a higher share in Ukraine (66.6%), whereas in the Republic of Moldova, urban areas are the majority (51.6%). At the organizational level, respondents predominantly come from the private sector (70.6%), particularly in the Republic of Moldova (77.6%), whereas in Romania, the public sector accounts for a higher share (33.2%). By organization size, the 10–49-employee category is the most frequent at the total level (36.8%), with a higher share in Ukraine (42.0%), followed by organizations with fewer than 9 employees (31.9%). The category of 50–249 employees accounts for 19.1% of the total, and large organizations (over 250 employees) account for 12.2%, with higher shares in Romania (18.2%) and the Republic of Moldova (12.8%) than in Ukraine (4.9%).

**Table 4:** Sample structure.

Variable	Category	Total (*N* = 1,007)	Romania (*n* = 407)	Ukraine (*n* = 350)	Republic of Moldova (*n* = 250)
Age	Under 25	536 (53.2%)	281 (69.0%)	85 (24.3%)	170 (68.0%)
	Between 26 and 45	268 (26.6%)	83 (20.4%)	136 (38.9%)	49 (19.6%)
	Between 46 and 60	195 (19.4%)	41 (10.1%)	129 (36.9%)	25 (10.0%)
	Over 60	8 (0.8%)	2 (0.5%)	0 (0.0%)	6 (2.4%)
Sex	Female	785 (78.0%)	322 (79.1%)	273 (78.0%)	190 (76.0%)
	Male	220 (21.8%)	83 (20.4%)	77 (22.0%)	60 (24.0%)
	I do not declare	2 (0.2%)	2 (0.5%)	0 (0.0%)	0 (0.0%)
Residence environment	Rural	578 (57.4%)	224 (55.0%)	233 (66.6%)	121 (48.4%)
	Urban	429 (42.6%)	183 (45.0%)	117 (33.4%)	129 (51.6%)
Organization type	Private	713 (70.8%)	270 (66.3%)	249 (71.1%)	194 (77.6%)
	Public	278 (27.6%)	135 (33.2%)	92 (26.3%)	51 (20.4%)
	Mixed / NGOs / Other	16 (1.6%)	2 (0.5%)	9 (2.6%)	5 (2.0%)
Organization size	Less than 9 employees	321 (31.9%)	118 (29.0%)	123 (35.1%)	80 (32.0%)
	10–49 employees	371 (36.8%)	142 (34.9%)	147 (42.0%)	82 (32.8%)
	50–249 employees	192 (19.1%)	73 (17.9%)	63 (18.0%)	56 (22.4%)
	More than 250 employees	123 (12.2%)	74 (18.2%)	17 (4.9%)	32 (12.8%)

In Romania, the most represented areas are trade (22.1%), marketing/sales (11.3%), and consultancy (10.8%), followed by construction/real estate (8.1%). In Ukraine, the structure is more concentrated in trade (27.7%) and marketing/sales (18.3%), whereas construction/real estate (15.1%) has a higher share than in other countries; consultancy accounts for 12.0%. In the Republic of Moldova, the dominant areas are trade (24.8%) and consultancy (12.8%), followed by marketing/sales (10.8%), indicating a profile relatively more oriented towards professional services than in Ukraine, where the transactional area and construction are more strongly emphasized.

In Romania (*n* = 407), the sample is dominated by financial-accounting functions (accountant/economist: 53.3%), followed by management (8.4%) and students (8.6%), while the administrative/civil servant category has a lower share (2.7%). In Ukraine (*n* = 350), the profile is similar to the financial-accounting core (52.0%), complemented by a relatively higher share of technical/engineering functions (9.4%) and associates/shareholders (7.1%), with management accounting for 5.7%. In the Republic of Moldova (*n* = 250), the share of accountant/economist is lower (40.0%), and the management segment is more pronounced (14.0%), followed by technical/engineering (6.4%) and associate/shareholder (5.6%).

In the distributional *χ*^2^ analysis (*N* = 1,007), with Holm adjustment for multiple comparisons, sample-level variation was observed for 8/11 items. Full details of frequencies and *χ*^2^ values are presented in Table 2. For the two ordinal 1–5 items (ITM_4 and ITM_11), these *χ*^2^ results refer to full response-category distributions, whereas the Kruskal-Wallis results in Table 5 were treated as the primary ordinal analyses. The significant results are: ITM_5, victimization, (*χ*^2^ = 32.552; pHolm = 0.000016; V = 0.127), ITM_2, financial distortions, (*χ*^2^ = 31.147; pHolm = 0.000029; *V* = 0.124), ITM_4, fraud-related/unethical proposal frequency, (*χ*^2^ = 39.142; pHolm = 0.000042; *V* = 0.139), ITM_9, fraud typology, (*χ*^2^ = 58.158; pHolm = 0.002361; *V* = 0.170), ITM_11, perceived gift/hospitality acceptance frequency, (*χ*^2^ = 25.880; pHolm = 0.007708; *V* = 0.113), ITM_6, reporting channel (*χ*^2^ = 16.722; pHolm = 0.013134; *V* = 0.091), ITM_8, colleague practices (*χ*^2^ = 14.790; pHolm = 0.025788; *V* = 0.086) and ITM_7, conditional acceptance/refusal under superior pressure (*χ*^2^ = 14.215; pHolm = 0.026561; *V* = 0.084). ITM_10 does not remain significant after correction (*χ*^2^ = 14.763; pHolm = 0.066551; *V* = 0.086), and ITM_1 (χ^2^ = 6.428; pHolm = 0.338800; *V* = 0.056) and ITM_3 (*χ*^2^ = 2.962; pHolm = 0.564121; *V* = 0.038) indicate the absence of statistically significant differences among surveyed national subsamples. Overall, the effect sizes are small to moderate (*V* = 0.084–0.170), with the most pronounced effect on ITM_9 (*V* = 0.170), suggesting sample-level variation among respondent groups, especially in fraud typology and experiential dimensions such as exposure/victimization (ITM_2, ITM_5).

**Table 5:** Kruskal–Wallis tests and effect size.

Item	H (Kruskal–Wallis)	*p*	*ε* ^2^	Median Romania	Median Ukraine	Median Republic of Moldova
ITM_4	31.228	< 0.000001	0.029	1.0	1.0	1.0
ITM_11	19.997	0.000045	0.018	1.0	2.0	1.0

**Table 6:** Pairwise *post hoc* for ITM_4 (Mann–Whitney U; Holm adjusted p).

Countries/pair	*U*	*p*	p (Holm)	r (rank-biserial)
Romania vs. Ukraine	57,990.0	<0.000001	0.000001	0.186
Romania vs. the Republic of Moldova	50,013.5	0.650993	0.650993	0.017
Ukraine vs. the Republic of Moldova	51,499.0	0.000030	0.000060	−0.177

To clarify the direction of the differences, among surveyed respondents from Ukraine, accounting distortions (27%) and personal victimization (14.6%) were reported most frequently, together with a higher share of high-frequency gift/hospitality acceptanceresponses (13%, categories 4–5). Among respondents from Romania, the frequencies of distortions (16%) and gifts (7%) were lower, and the share of “Never” responses for victimization was higher (59.7%). The respondent group from the Republic of Moldova was positioned between Ukraine and Romania for personal victimization (13.2%), but recorded the highest share of indirect victimization, measured as knowing colleagues who had been victimized (46.4%), and the highest rate of reporting directly to the manager (68%).

According to Tables 5–7, for ITM_4, the global test indicates significant differences among surveyed national subsamples (*H* = 31.228; *p* = 1.66 × 10^−7^; *ε*^2^ = 0.029). Post-hoc shows significant differences between Romania and Ukraine (pHolm = 1.06 × 10^−6^) and between Ukraine and the Republic of Moldova (pHolm = 6.05 × 10^−5^), while the Romania and Republic of Moldova respondent groups did not differ significantly (pHolm = 0.651). For ITM_11, the overall differences are also significant (H = 19.997; *p* = 4.55 × 10^−5^; *ε*^2^ = 0.018), and post-hoc indicates differences between Romania and Ukraine (pHolm = 2.49 × 10^−5^) and between Ukraine and the Republic of Moldova (pHolm = 0.044), with no significant differences between Romania and the Republic of Moldova (pHolm = 0.096). Overall, the effect sizes are small (*ε*^2^ = 0.018–0.029), but remain consistent after correction, suggesting that, within this non-probabilistic sample, the Ukrainian respondent group displayed a distinct ordinal response profile relative to the other two respondent groups.

**Table 7:** Pairwise *post hoc* for ITM_11 (Mann–Whitney *U*; Holm adjusted *p*).

Countries/pair	*U*	*p*	p (Holm)	r (rank-biserial)
Romania vs. Ukraine	59,340.5	0.000008	0.000025	0.167
Romania vs. the Republic of Moldova	47,504.5	0.0956676	0.0956676	0.066
Ukraine vs. the Republic of Moldova	48,122.0	0.0220251	0.0440503	−0.100

For ITM_4, the medians were identical across the three surveyed national subsamples (1.0), so the Kruskal-Wallis result reflects differences in the upper tail of the distribution. Respondents in Ukraine reported answers “3–5” more often (28.6%) than respondents in Romania (18.2%) or the Republic of Moldova (17.2%). For ITM_11, the median was higher among surveyed respondents from Ukraine (2.0) than among respondents from Romania and the Republic of Moldova (1.0 in both groups), alongside a higher proportion of “often/very often” responses in Ukraine (12.9%) than in Romania (7.1%) and the Republic of Moldova (10.0%).

After adjustment for age group, gender, residence environment, organization type, and organization size, several national-subsample contrasts persisted. Compared with respondents from Romania, respondents from Ukraine had higher adjusted odds of reporting more frequent fraud-related/unethical proposals (ITM_4: OR = 2.28, 95% CI 1.63–3.18), higher perceived workplace gift/hospitality acceptance frequency (ITM_11: OR = 1.77, 95% CI 1.28–2.43), a higher likelihood of reporting no action rather than notifying management (ITM_6: OR = 3.82, 95% CI 1.65–8.85), and higher odds of selecting a non-refusal response to superior-requested fraud (ITM_7: OR = 2.41, 95% CI 1.38–4.20).

Compared with respondents from Romania, respondents from the Republic of Moldova had higher adjusted odds of personal victimization (ITM_5: OR = 2.38, 95% CI 1.36–4.16), indirect victimization (ITM_5: OR = 2.51, 95% CI 1.76–3.60), non-refusal responses to superior-requested fraud (ITM_7: OR = 2.22, 95% CI 1.29–3.82), and perceived colleague involvement (ITM_8: OR = 2.82, 95% CI 1.63–4.87). Other adjusted national-subsample contrasts were attenuated and were not retained after Holm correction. These findings indicate that some observed differences were not fully explained by the measured sample-composition variables, although they remain exploratory sample-level associations.

For ITM_5, a supplementary multinomial logistic regression model retaining all three response categories yielded substantively similar national-subsample contrasts. For the supplementary ITM_5 multinomial sensitivity model, Holm adjustment was applied across the four national-subsample contrasts reported for the two non-reference outcome categories. Compared with respondents from Romania, respondents from Ukraine had higher adjusted odds of indirect victimization (OR = 1.60, 95% CI 1.12–2.30, Holm-adjusted *p* = 0.031) and personal victimization (OR = 1.78, 95% CI 1.05–3.01, Holm-adjusted *p* = 0.032). Respondents from the Republic of Moldova also had higher adjusted odds of indirect victimization (OR = 2.54, 95% CI 1.78–3.64, Holm-adjusted *p* < 0.001) and personal victimization (OR = 2.01, 95% CI 1.18–3.45, Holm-adjusted *p* = 0.031). This sensitivity analysis indicates that the ITM_5 findings were not an artifact of estimating two separate binary contrasts.

For ITM_6, a supplementary penalized multinomial logistic regression model retaining all three response categories yielded results consistent with the binary contrasts. With notifying management as the reference outcome, respondents from Ukraine had higher adjusted odds of external/formal reporting than respondents from Romania (OR = 1.42, descriptive bootstrap 95% CI 1.01–2.04) and higher adjusted odds of reporting no action (OR = 3.67, descriptive bootstrap 95% CI 1.61–8.75). The Republic of Moldova contrast was close to null for external/formal reporting (OR = 0.87, descriptive bootstrap 95% CI 0.61–1.23), while the no-action contrast should be interpreted cautiously because this category contained very few observations in that subsample.

For ITM_7, the supplementary penalized multinomial model separated the two categories combined in the main binary non-refusal contrast. With refusal (“No”) as the reference outcome, respondents from Ukraine had higher adjusted odds of both “Depends on the situation” (OR = 2.30, descriptive bootstrap 95% CI 1.20–4.54) and “Yes, if I also benefit” (OR = 2.20, descriptive bootstrap 95% CI 1.29–4.20) compared with respondents from Romania. For respondents from the Republic of Moldova, the contrast was more evident for “Yes, if I also benefit” (OR = 2.57, descriptive bootstrap 95% CI 1.38–5.03) than for “Depends on the situation” (OR = 1.61, descriptive bootstrap 95% CI 0.71–3.02). This sensitivity analysis supports the main non-refusal finding while showing that the two response categories should not be treated as psychologically identical.

For ITM_8, the sensitivity model, excluding uncertain or non-response categories and contrasting only affirmative responses with explicit “No” responses, yielded substantively unchanged results. Compared with respondents from Romania, respondents from Ukraine had higher adjusted odds of reporting perceived colleague involvement (OR = 2.12, 95% CI 1.19–3.79), and respondents from the Republic of Moldova also had higher adjusted odds (OR = 2.84, 95% CI 1.64–4.90). This confirms that the ITM_8 association was not driven by combining “Do not know” or other uncertain responses with explicit negative responses.

Proportional-odds diagnostics, summarized in Supplementary Table 5, showed no strong evidence of material threshold variation for the national-subsample contrasts in either ITM_4 or ITM_11. Some threshold variation was observed for organization-size covariates in the ITM_11 model; therefore, multinomial logistic regression models were estimated as sensitivity analyses. These sensitivity models supported the substantive interpretation of the main national-subsample contrasts: the Ukraine contrast remained positive for ITM_4 and ITM_11 across response categories, whereas the Republic of Moldova contrasts were less consistent and should be interpreted cautiously.

For the original ITM_9 item, the results were *χ*^2^ = 58.158, *p* = 0.000295, and Cramér’s *V* = 0.170. For the four-category aggregated version, the result remained significant: *χ*^2^ = 37.307, df = 6, asymptotic *p* = 1.53 × 10^−6^, Monte Carlo *p* = 0.000200, Cramér’s *V* = 0.136. This sensitivity check suggests that the association between national subsample membership and perceived fraud typology was unlikely to be driven solely by sparse response categories.

According to Figure 1, the MCA projection onto the first two dimensions (Dim1–Dim2) highlights a two-cluster structure. Cluster 1 (*n* = 866) represents the dominant lower-exposure/refusal-dominant response profile, while Cluster 2 (*n* = 141) represents a smaller higher-exposure/non-refusal-enriched response profile. The first two dimensions together account for 27.99% of the total inertia, which is typical for MCA with categorical data, where variation is distributed across several dimensions. Thus, Dim1 and Dim2 provide a two-dimensional visualization of response-pattern proximity but do not fully capture the categorical data’s multidimensional structure. Cluster labels were assigned descriptively based on the modal and over-represented response categories across the six MCA input items, as reported in Supplementary Table 4.

**Figure 1:**
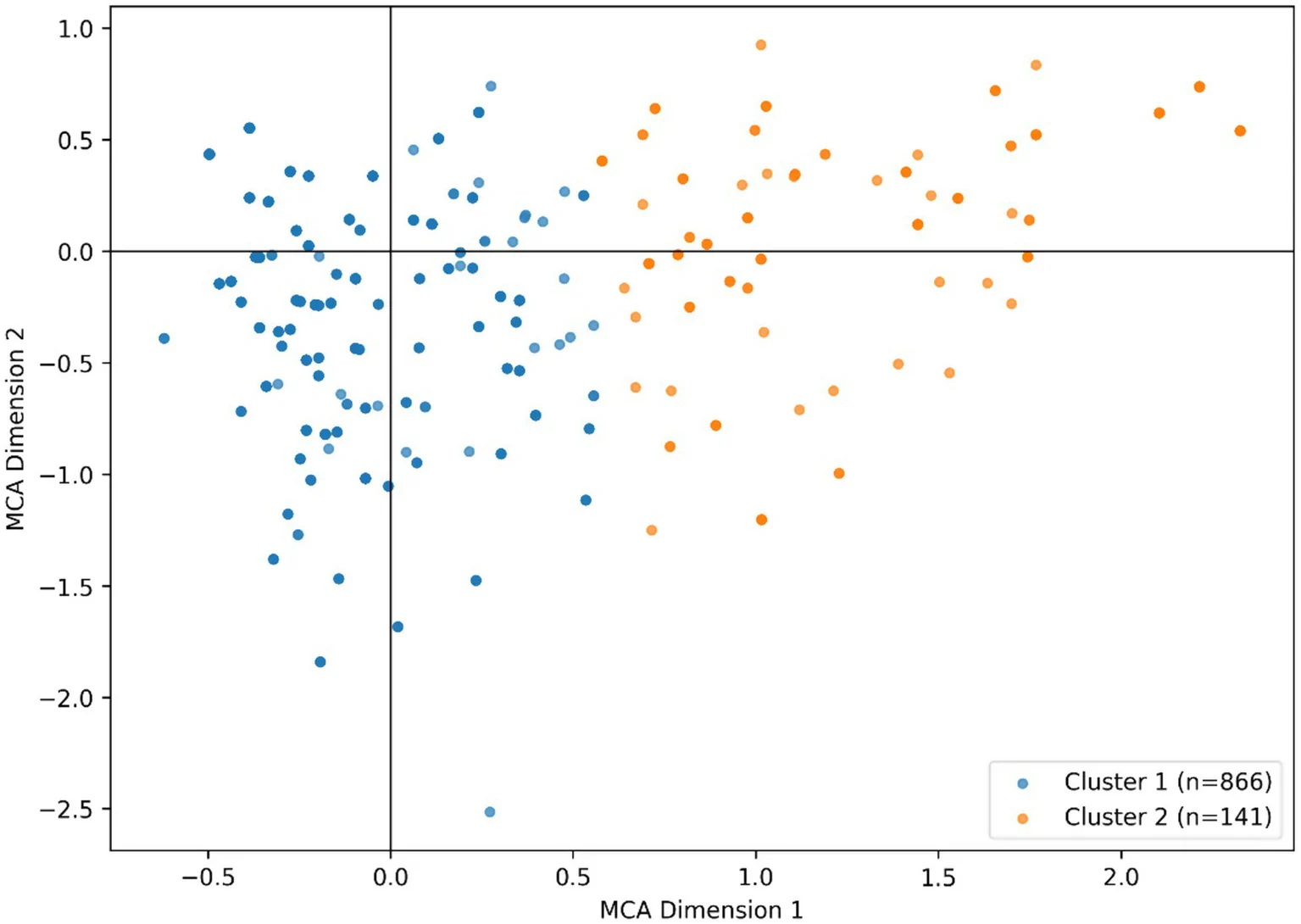
MCA projection (Dim 1–Dim 2) with clusters. Source: Authors’ analysis.

The frequencies in Table 8 indicate the number of respondents from each national subsample in each profile: Romania: C1 = 368, C2 = 39; Ukraine: C1 = 290, C2 = 60; Republic of Moldova: C1 = 208, C2 = 42. The majority of respondents from all countries are found in Cluster 1, and Cluster 2 was proportionally more frequent in Ukraine (17.1%) and the Republic of Moldova (16.8%) than in Romania (9.6%).

**Table 8:** Distribution of clusters by national subsample.

Country	Cluster 1	Cluster 2	Total
Republic of Moldova	208 (83.2%)	42 (16.8%)	250
Romania	368 (90.4%)	39 (9.6%)	407
Ukraine	290 (82.9%)	60 (17.1%)	350
Total	866	141	1,007

The association test (Table 9) indicates a significant relationship between country and cluster membership (*χ*^2^ = 11.095; df = 2; *p* = 0.003897), but the effect size is small (Cramér’s *V* = 0.105), which shows that cluster proportions differed across surveyed national subsamples, but the magnitude of the difference was small. In other words, the typologies appear in all national subsamples surveyed, but in different proportions.

**Table 9:** Association between national subsample membership and cluster membership.

Association test	Value
*χ* ^2^	11.095
df	2
*p*	0.003897
Cramér’s V	0.105

## 4 Discussion

The results support the existence of statistically significant sample-level differences among the surveyed national subsamples in several experiential and organizational dimensions of occupational fraud, although most effect sizes were small. The *χ*^2^ analysis with Holm correction shows that 8 out of 11 items (ITM_2, ITM_4, ITM_5, ITM_6, ITM_7, ITM_8, ITM_9, ITM_11) differ significantly across national subsamples surveyed, with small to moderate effect sizes (Cramér’s *V* = 0.084–0.170). The most consistent differences occur for direct or proximate experiences of fraud (ITM_5 - victimization; ITM_4 - proposals to engage in potentially fraudulent or unethical activities; ITM_2 - misstatements/violations of financial statements), suggesting that perceived exposure and fraud-related experiences vary across surveyed respondent groups and organizational profiles, although the magnitude of these differences remains limited. At the same time, ITM_1 (fraud vs. corruption) and ITM_3 (stated/perceived existence of anti-fraud procedures) do not differ significantly across national subsamples surveyed, indicating a relatively similar core of general perceptions and reporting of the presence of procedures, although their effectiveness may vary. This pattern is consistent with ACFE’s observations that internal control weaknesses and control circumvention are common factors in occupational fraud, and formal measures may exist without eliminating perceived risks.

Interpreted through the Fraud Triangle and Fraud Diamond frameworks, these findings suggest that the observed response patterns are more closely related to perceived opportunity, informal normalization, and reporting conditions than to a single national effect. The adjusted persistence of selected associations suggests that perceived opportunity and informal-norm indicators are not reducible only to the measured demographic and organizational composition of the subsamples. Items that capture misstatements or violations in financial statements, fraud-related/unethical proposals, and victimization are consistent with the opportunity component of the Fraud Triangle because they point to perceived weaknesses in controls, supervision, or organizational safeguards. The differences observed in non-refusal responses to superior-requested fraud and perceived colleague involvement are more closely linked to rationalization and informal norms, suggesting that some respondents may perceive fraudulent conduct as situationally negotiable or normalized in the workplace. The Fraud Diamond also helps interpret these findings cautiously: although individual capability was not directly measured, organizational position, reporting channels, and perceived control environments may condition whether opportunities can be exploited or resisted. These findings should therefore be interpreted as exploratory, sample-level associations rather than as causal effects or representative estimates of the employed populations of the three countries.

Specifically for the ordinal items, Kruskal-Wallis and post-hoc tests clarified where the differences lie: both ITM_4 (frequency of proposals to engage in potentially fraudulent or unethical activities) and ITM_11 (frequency of perceived gift/hospitality acceptance) show significant overall differences across national subsamples surveyed, and pairwise analysis systematically indicates contrasts involving Ukraine versus the other two respondent groups. At the same time, the Romania and Republic of Moldova respondent groups did not differ significantly on these scales. The effect sizes (ε^2^) are small but robust, which is typical in comparative research on sensitive behaviors/attitudes, where underreporting and social desirability bias can attenuate observable differences, even when differences remain statistically significant.

The practical significance of these findings should therefore be interpreted proportionately. The Cramér’s V values for categorical items and the *ε*^2^ values for ordinal items indicate mostly small or small-to-moderate associations, meaning that the observed differences are useful for identifying areas of organizational attention rather than for ranking countries or deriving direct policy prescriptions. Accordingly, the most defensible practical contribution of the study is to indicate where reporting channels, internal controls, ethical climate, and perceived exposure patterns may require further organizational assessment.

The adjusted outcome models directly addressed the concern that differences between surveyed national subsamples could be confounded by age, residence, organizational type, and organization size. After adjustment, several contrasts persisted, especially for Ukraine on fraud-related/unethical proposal frequency, perceived gift/hospitality acceptance practices, no-action reporting response, and non-refusal responses to superior-requested fraud, and for the Republic of Moldova on personal and indirect victimization, non-refusal responses to superior-requested fraud, and perceived colleague involvement. At the same time, some descriptive differences were attenuated after adjustment, confirming that sample composition matters. These results therefore provide more defensible evidence than crude national-subsample comparisons alone, but they remain exploratory associations within a non-probabilistic sample.

Research among internal auditors in Indonesian public universities has linked whistleblowing intentions to ethical awareness and attitudes towards reporting, with a moderating role for perceived ethical climate (41). Complementary conceptual work uses the Three Lines Model to clarify organizational roles and responsibilities within whistleblowing frameworks (43). The reporting-channel item shows that differences are not limited to whether fraud is perceived but also concern how respondents imagine institutional responses and disclosures. The higher adjusted odds of a no-action response among respondents from Ukraine, and the differences in manager-oriented or external reporting preferences, suggest that whistleblowing behavior may depend on perceived protection, trust in internal channels, and expectations that reports will lead to corrective action. This supports the view that anti-fraud governance is not only a technical control problem, but also a climate problem: formal procedures may exist, yet employees may remain reluctant to report if they expect retaliation, inaction, or weak organizational feedback. These mechanisms were not measured directly in the present survey and should therefore be regarded as hypotheses for future research rather than as explanatory findings of the current study.

We explicitly addressed a frequent methodological criticism in *χ*^2^ studies: the ITM_9 item has many categories (14), which can generate rare cells. The sensitivity analysis based on the four-category version of ITM_9, defined as the three most frequent categories plus “Other/rare”, maintained statistical significance. It preserved a comparable effect (V decreasing but remaining within a narrow range), indicating that the rarity of some categories does not artificially produce differences in perceived fraud typology across surveyed national subsamples but rather reflects a robust pattern. At the same time, the typology analysis (MCA followed by k-means clustering) offered a complementary perspective. A two-cluster solution was retained because it showed the strongest diagnostic profile (silhouette = 0.422; bootstrap ARI = 0.944; cluster sizes: C1 = 866, C2 = 141). Cluster 1 represented the dominant lower-exposure/refusal-dominant response profile, while Cluster 2 represented a smaller higher-exposure/non-refusal-enriched profile. Cluster membership was significantly associated with national subsample membership, although the effect was small (*χ*^2^ = 11.095, df = 2, *p* = 0.003897, Cramér’s *V* = 0.105). Thus, the typology analysis complements the item-level interpretation by showing that aggregate response profiles are present across all three surveyed subsamples, albeit in different proportions. The methodological justification of MCA for categorical data is consistent with standard definitions of the method. Although statistically significant, most associations had small effect sizes; therefore, their practical meaning lies primarily in identifying areas for further organizational assessment rather than in supporting direct policy prescriptions.

A study of Indonesian government internal auditors reported positive associations between the internal audit function and both internal control and governance (44). Overall, the results outline cautious practical implications for internal control and compliance practices: the dimensions where differences are more pronounced (exposure/victimization, types of fraud, perceived gift/hospitality acceptance practices, reactions in case of victimization) are consistent with the areas where organizational interventions (reporting channels, management review, controls, ethical culture) are frequently described in the applied literature as relevant for detecting and limiting losses, especially through “tips/whistleblowing” mechanisms.

Given the generally small effect sizes, the practical implications should be interpreted cautiously and as directions for further organizational attention rather than as direct policy prescriptions. The findings suggest that reporting channels, management responsiveness, non-retaliation guarantees, ethics training, and internal-control procedures may be relevant areas for future intervention or closer organizational assessment. However, because the study is exploratory, non-probabilistic, and based on self-reported survey items, these implications should be treated as hypothesis-generating and should be tested in representative or organizationally embedded studies before being translated into policy recommendations. Table 10 presents the research hypotheses, the supporting evidence from the specialized literature, the statistical analyses used, and the level of empirical support, taking into account both statistical significance and effect size.

**Table 10:** Correspondence matrix between hypotheses, literature, and results.

Hypothesis	Authors– literature review	Statistical analysis used in the study	Status
H1: Differences among surveyed national subsample sregarding exposure/perception of fraud (ITM_2, ITM_4, ITM_5).	Wolfe and Hermanson (4) and ACFE (13).	*χ*^2^; Cramér’s *V*; Holm adjustment (ITM_2, ITM_5); Kruskal–Wallis and post-hoc (Mann–Whitney U pairwise, Holm) (ITM_4).	Exploratory supported; statistically significant differences were observed across surveyed national subsamples, with small-to-moderate effect sizes.
H2: Differences among surveyed national subsamples regarding attitudes/acceptability (ITM_7, ITM_8).	Ajzen (38) and Wolfe and Hermanson (4).	*χ*^2^; Cramér’s *V*; Holm adjustment (ITM_7, ITM_8).	Exploratory supported; statistically significant differences were observed across surveyed national subsamples, with small effect sizes.
H3: Differences among surveyed national subsamples regarding reporting/management and associated practices (ITM_6, ITM_10, ITM_11).	Committee of Sponsoring Organizations of the Treadway Commission (39) and ACFE (13).	*χ*^2^; Cramér’s *V*; Holm adjustment (ITM_6, ITM_10); Kruskal–Wallis and post-hoc (Mann–Whitney U pairwise, Holm) (ITM_11).	Partially and exploratory supported; ITM_6 and ITM_11 were significant, whereas ITM_10 did not remain significant after Holm correction; effect sizes were small.
H4: After adjustment for respondent and organizational characteristics, national subsample membership remains associated with selected fraud-related outcomes.	Hogan et al. (8), Beasley et al. (6), Hamed (27), Maulidi et al. (28), Novatiani et al. (29), Gherai et al. (26), and Achmad et al. (32).	Covariate-adjusted ordinal and binary logistic regression models; Romania as reference category; covariates: age group, gender, residence environment, organization type, and organization size; ORs, 95% CIs, raw *p*-values, and Holm-adjusted p-values.	Partially supported; several adjusted contrasts persisted, but the findings are interpreted as exploratory adjusted associations rather than causal or nationally representative effects.
Robustness: ITM_9 – differences in fraud types are not the artifact of rare categories (14 categories).	Lenz (34)	*χ*^2^; Cramér’s *V* (ITM_9 original vs. three most frequent categories plus Other/rare, yielding a four-category version).	Supported; results remained significant after category aggregation, with a small-to-moderate effect size.
Typologies: different aggregate response profiles among surveyed national subsamples.	Benzécri (36) and Greenacre (37).	MCA (Dim1–Dim2) and *k*-means clustering (*k* = 2); *χ*^2^ Country×Cluster; Cramér’s *V*.	Exploratorily supported; cluster membership was associated with national subsample membership, but the effect size was small.

This study faces several constraints. First, the data are self-reported and therefore vulnerable to social desirability bias and selective underreporting of sensitive incidents. Second, the cross-sectional design supports only associative, not causal, interpretations. Third, national subsamples differ in age structure, which may shift prevalence estimates even after multivariate controls. Fourth, some items with many response categories (e.g., ITM_9) produce sparse cells; robustness checks that aggregate categories mitigate this issue but do not eliminate it. Fifth, voluntary online recruitment via Google Forms entails self-selection: employees with limited Internet access or low interest in fraud topics may be under-represented. Sixth, the questionnaire captured item-level indicators of exposure, perceived norms, reporting intentions, and conditional acceptance/refusal responses, rather than validated multi-item psychometric scales. As a result, internal consistency could not be assessed for most thematic domains, and the findings should be interpreted as response-level evidence rather than as a measurement of latent constructs. The victimization item did not explicitly restrict respondents to workplace or occupational settings. Therefore, personal and indirect victimization responses may include experiences of fraud beyond strictly occupational fraud, and these findings should be interpreted accordingly. In particular, ITM_1 and ITM_7 have wording-response limitations: ITM_1 does not measure the degree of perceived closeness between occupational fraud and corruption, and ITM_7 conflates compliance under superior pressure with a personal-benefit condition in a single response option. The binary non-refusal contrast for ITM_7 combines two conceptually distinct response categories (“Depends on the situation” and “Yes, if I also benefit”), and the resulting odds ratio should therefore be interpreted as a broad forced-choice response pattern rather than as a homogeneous attitudinal construct or as a direct measure of the probability of compliance. Consequently, the findings should be interpreted as exploratory evidence based on individual survey items, not as estimates derived from validated latent constructs. Seventh, although the questionnaire was translated and piloted, formal back-translation was not performed, so subtle semantic differences between languages cannot be ruled out. For this reason, cross-subsample comparisons should be interpreted with caution, as item-level comparisons are based on harmonized survey wording rather than comparisons derived from formally invariant psychometric scales. Eighth, post-stratified weighting was not feasible; the over-representation of respondents under 25 years could bias frequency estimates. Ninth, the adjusted outcome models rely exclusively on self-reported survey variables and were estimated during a period of macroeconomic and geopolitical turbulence (e.g., the war in Ukraine), factors that may shape perceptions in ways that are difficult to disentangle from organizational effects. Tenth, the reporting-channel item offered a limited set of response options and may not have captured the full diversity of institutional reporting pathways available across the three countries, such as internal audit, compliance officers, ethics hotlines, police or prosecutorial authorities, anonymous whistleblowing systems, or external auditors. Therefore, findings related to reporting behavior should be interpreted as indicative rather than exhaustive. Moreover, the external-reporting option may not have had identical institutional meaning across the three national contexts, because OLAF and the Fiscal State Service may represent different practical reporting pathways for respondents. Finally, the study did not directly measure psychological safety, retaliation fears, mental health, occupational stress, or health outcomes; therefore, the public-health interpretation should be understood as an organizational-health framing rather than as direct evidence of health effects.

Future research directions can strengthen these results by: (i) sampling closer to representativeness criteria by sectors and occupations; (ii) more granular measurement of opportunities and internal controls (multi-item scales); (iii) longitudinal analyses to capture changes over time and intervention effects; (iv) inclusion of objective organizational indicators (e.g., existence of internal audit, type of control, existence of reporting channels and their degree of use); and (v) extended comparisons to other countries in the region to test the stability of the identified typologies. These extensions would allow the transformation of comparative conclusions into more precise operational recommendations for governance and compliance, calibrated to the context-specific risks observed in comparable samples.

## 5 Conclusion

In line with the literature on occupational fraud, the findings of this study support the idea that risk is determined not by a single factor but by a combination of organizational opportunity, internal norms, and the reporting climate. Within this non-probabilistic sample, these dimensions varied across surveyed respondent groups rather than supporting direct country-level generalizations. The survey-based comparative results indicate that, among respondents from Romania, Ukraine, and the Republic of Moldova, there is a relatively similar core of general perceptions (e.g., at the level of conceptual definition/framing or the declared existence of procedures). However, differences become visible when the analysis shifts to dimensions closer to practice: exposure/victimization, fraud typologies, reporting/reactions, and related workplace practices, including perceived gift/hospitality acceptance.

At a concrete level, the observed differences should be interpreted as patterns among surveyed respondents rather than as nationally representative estimates. Within the adjusted analyses, the Ukrainian respondent group was distinguished mainly by higher fraud-related/unethical proposal frequency, perceived gift/hospitality acceptance, a greater likelihood of reporting no action rather than notifying management, and non-refusal responses to superior-requested fraud. The Republic of Moldova respondent group was more clearly distinguished by personal and indirect victimization, non-refusal responses to superiors’ requests for fraud, and perceived involvement of colleagues. These patterns remain informative for exploratory comparison, but they should be interpreted in light of the unequal age, gender, occupational, and recruitment-channel structure of the sample. Accordingly, the study does not support causal or population-level conclusions, but provides exploratory evidence on response patterns within the surveyed groups.

Overall, these differences suggest that anti-fraud interventions may benefit from sensitivity to the respondent profiles and organizational contexts observed in each subsample, rather than assuming a uniform pattern across settings. This interpretation is consistent with the Fraud Triangle/Fraud Diamond perspective, but the present study supports it only indirectly through survey-based indicators of perceived exposure, informal norms, and reporting behavior. From the perspective of preventing and reducing occupational fraud, the implications are oriented towards measures with recognized efficiency in the applied literature: strengthening internal controls (with emphasis on separation of duties and independent verifications), increasing the perceived probability of detection/sanctioning (through risk-oriented internal audit and monitoring), standardizing reporting channels (including protected whistleblowing mechanisms) and interventions on the ethical climate (tone at the top, training applied to dilemmas and scenarios). An essential practical direction is to shift the emphasis from the formal existence of procedures to their actual functioning: trust in reporting, protection of reporters, consistency of sanctions, and organizational feedback following notifications. These recommendations should be interpreted as directions suggested by the observed survey patterns and by prior literature, rather than as direct evidence of intervention effectiveness.

From an occupational and organizational health perspective, fraud prevention appears connected to workplace governance, reporting culture, perceived employee protection, and ethical leadership. Measures aimed at improving trust in reporting channels, protecting whistleblowers, and strengthening ethical leadership are plausible areas for future organizational intervention, but the present data do not allow direct conclusions about intervention effectiveness or population-level policy impact.

## Data Availability

The data analyzed in this study is subject to the following licenses/restrictions: the de-identified dataset and analysis code are available from the corresponding author upon reasonable request. Open release of the raw dataset is restricted because the survey includes sensitive self-reported information on fraud exposure, reporting behavior, and workplace practices. Any shared dataset will exclude free-text responses and technical metadata. Requests to access these datasets should be directed to LM-D, lucia.danila@usm.ro.
