# Supplementary material for: Survey-based evidence on occupational fraud exposure, reporting behavior, and informal norms in Romania, Ukraine, and the Republic of Moldova
Source: Front Public Health. 2026 Sep 25;14:1923314. doi: 10.3389/fpubh.2026.1923314 (PMC13649425; doi:10.3389/fpubh.2026.1923314)
Supplement: Supplementary file 1 [file Table_1.docx]

# Supplementary material

**Supplementary Table 1**. Correspondence of codes with questionnaire items

| Item code | Item wording | Analytical domain / item-level indicator | Response format | Coding / analysis |
| --- | --- | --- | --- | --- |
| ITM_1 | In your opinion, how closely related are occupational fraud and corruption? | Perceived association between occupational fraud and corruption | Nominal, 3 categories | Yes / No / Do not know or no answer; analyzed as categorical  Analyzed as categorical; not interpreted as degree of closeness |
| ITM_2 | At the company/institution where you work, have you encountered cases where significant misstatements or other violations of financial statements have been committed? | Workplace exposure to accounting distortions or financial statement violations | Nominal, 3 categories | Yes / No / Do not know or no answer; analyzed as categorical |
| ITM_3 | Does the company/institution where you work have procedures for identifying and preventing fraud? | Perceived existence of anti-fraud procedures | Nominal, 3 categories | Yes / No / Do not know or no answer; analyzed as categorical |
| ITM_4 | In the last three years, how often have you been asked to participate in activities that could be considered fraudulent or unethical? | Frequency of exposure to potentially fraudulent or unethical activity proposals | Ordinal 1-5 scale | Higher values indicate higher frequency; analyzed with Kruskal-Wallis tests and as an ordinal outcome |
| ITM_5 | Have you been the victim of fraud committed by someone you trusted? | Personal or indirect victimization experience | Nominal, 3 categories | Never / No, but I know colleagues who were / Yes; analyzed as categorical |
| ITM_6 | If you became aware of occupational fraud in your organization, whom would you contact first? | Reporting intention / first reporting channel | Nominal, 3 categories | Manager / OLAF or Fiscal State Service / Nothing; analyzed as categorical |
| ITM_7 | If a superior asked you to participate in a fraudulent act, how likely would you be to comply? | Forced-choice response to superior-requested fraud | Nominal, 3 categories | No / Depends on the situation / Yes, if I also benefit; analyzed as categorical. In adjusted models, "Depends" and "Yes, if I also benefit" were combined as non-refusal vs refusal; not interpreted as a continuous likelihood measure or as a pure profit-motive scale. |
| ITM_8 | Have you observed colleagues engaging in practices that may be considered fraudulent or unethical? | Perceived informal workplace norms / colleague behavior | Nominal, 3 categories | Yes / No / Do not know or no answer; analyzed as categorical |
| ITM_9 | What are the most common types of fraud? | Perceived fraud typology | Nominal, multiple categories | Original 14 categories; sensitivity analysis used aggregation into three most frequent categories plus Other/rare, yielding a four-category version |
| ITM_10 | In your opinion, how should fraud risks be managed? | Preferred fraud-risk management response | Nominal, 4 categories | Risk assessment/control system / Reporting suspicious events / Preserving evidence / Other or rare; analyzed as categorical |
| ITM_11 | How often are gifts or hospitality from clients accepted in your workplace? | Perceived workplace gift/hospitality acceptance frequency | Ordinal 1-5 scale | Higher values indicate higher perceived frequency; analyzed with Kruskal-Wallis tests and as an ordinal outcome |

**Supplementary Table 2.** Full covariate-adjusted model estimates

| Outcome | Model | Level_or_event | N | Term | log_OR | OR | CI_low | CI_high | p |
| --- | --- | --- | --- | --- | --- | --- | --- | --- | --- |
| ITM4_fraud_related_unethical_proposal_frequency | Ordinal logistic regression | Higher fraud-related/unethical proposal frequency | 987 | country=Ukraine | 0.823078 | 2.277499 | 1.630594 | 3.18105 | 1.38E-06 |
| ITM4_fraud_related_unethical_proposal_frequency | Ordinal logistic regression | Higher fraud-related/unethical proposal frequency | 987 | country=Republic of Moldova | 0.065789 | 1.068002 | 0.757536 | 1.505708 | 0.707352 |
| ITM4_fraud_related_unethical_proposal_frequency | Ordinal logistic regression | Higher fraud-related/unethical proposal frequency | 987 | age_group=26-45 | -0.42916 | 0.651053 | 0.460247 | 0.920963 | 0.015298 |
| ITM4_fraud_related_unethical_proposal_frequency | Ordinal logistic regression | Higher fraud-related/unethical proposal frequency | 987 | age_group=46+ | -0.42759 | 0.652076 | 0.452297 | 0.940098 | 0.021968 |
| ITM4_fraud_related_unethical_proposal_frequency | Ordinal logistic regression | Higher fraud-related/unethical proposal frequency | 987 | gender=Male | 0.460048 | 1.58415 | 1.166228 | 2.151837 | 0.00324 |
| ITM4_fraud_related_unethical_proposal_frequency | Ordinal logistic regression | Higher fraud-related/unethical proposal frequency | 987 | residence=Urban | -0.11277 | 0.893355 | 0.681129 | 1.171707 | 0.415131 |
| ITM4_fraud_related_unethical_proposal_frequency | Ordinal logistic regression | Higher fraud-related/unethical proposal frequency | 987 | org_type=Public | -0.03271 | 0.967816 | 0.717885 | 1.304759 | 0.830055 |
| ITM4_fraud_related_unethical_proposal_frequency | Ordinal logistic regression | Higher fraud-related/unethical proposal frequency | 987 | org_size=10-49 | 0.107622 | 1.113627 | 0.821633 | 1.509391 | 0.487886 |
| ITM4_fraud_related_unethical_proposal_frequency | Ordinal logistic regression | Higher fraud-related/unethical proposal frequency | 987 | org_size=50-249 | -0.14475 | 0.865236 | 0.579408 | 1.292067 | 0.479248 |
| ITM4_fraud_related_unethical_proposal_frequency | Ordinal logistic regression | Higher fraud-related/unethical proposal frequency | 987 | org_size=250+ | -0.4257 | 0.65331 | 0.398476 | 1.071117 | 0.091486 |
| ITM11_gift_hospitality_acceptance_frequency | Ordinal logistic regression | Higher perceived gift/hospitality acceptance frequency | 987 | country=Ukraine | 0.568626 | 1.76584 | 1.281882 | 2.43251 | 0.000502 |
| ITM11_gift_hospitality_acceptance_frequency | Ordinal logistic regression | Higher perceived gift/hospitality acceptance frequency | 987 | country=Republic of Moldova | 0.277921 | 1.320382 | 0.952403 | 1.830536 | 0.095437 |
| ITM11_gift_hospitality_acceptance_frequency | Ordinal logistic regression | Higher perceived gift/hospitality acceptance frequency | 987 | age_group=26-45 | -0.07129 | 0.931192 | 0.672514 | 1.289368 | 0.667674 |
| ITM11_gift_hospitality_acceptance_frequency | Ordinal logistic regression | Higher perceived gift/hospitality acceptance frequency | 987 | age_group=46+ | 0.453937 | 1.574499 | 1.119382 | 2.214657 | 0.009111 |
| ITM11_gift_hospitality_acceptance_frequency | Ordinal logistic regression | Higher perceived gift/hospitality acceptance frequency | 987 | gender=Male | 0.415599 | 1.515277 | 1.130386 | 2.031223 | 0.005441 |
| ITM11_gift_hospitality_acceptance_frequency | Ordinal logistic regression | Higher perceived gift/hospitality acceptance frequency | 987 | residence=Urban | -0.2894 | 0.748714 | 0.576651 | 0.972116 | 0.029838 |
| ITM11_gift_hospitality_acceptance_frequency | Ordinal logistic regression | Higher perceived gift/hospitality acceptance frequency | 987 | org_type=Public | 0.291667 | 1.338657 | 1.007451 | 1.778749 | 0.044309 |
| ITM11_gift_hospitality_acceptance_frequency | Ordinal logistic regression | Higher perceived gift/hospitality acceptance frequency | 987 | org_size=10-49 | 0.057232 | 1.058902 | 0.782606 | 1.432743 | 0.710642 |
| ITM11_gift_hospitality_acceptance_frequency | Ordinal logistic regression | Higher perceived gift/hospitality acceptance frequency | 987 | org_size=50-249 | 0.035372 | 1.036006 | 0.712472 | 1.506455 | 0.853088 |
| ITM11_gift_hospitality_acceptance_frequency | Ordinal logistic regression | Higher perceived gift/hospitality acceptance frequency | 987 | org_size=250+ | 0.273573 | 1.314654 | 0.843395 | 2.049234 | 0.227072 |
| ITM5_personal_victimization | Binary logistic regression | Personal victimization vs never | 603 | const | -1.73839 | 0.175803 | 0.106504 | 0.290194 | 1.06E-11 |
| ITM5_personal_victimization | Binary logistic regression | Personal victimization vs never | 603 | country=Ukraine | 0.536631 | 1.710235 | 1.016114 | 2.87852 | 0.043369 |
| ITM5_personal_victimization | Binary logistic regression | Personal victimization vs never | 603 | country=Republic of Moldova | 0.867726 | 2.381488 | 1.363712 | 4.15886 | 0.002284 |
| ITM5_personal_victimization | Binary logistic regression | Personal victimization vs never | 603 | age_group=26-45 | -0.06615 | 0.935987 | 0.552236 | 1.586406 | 0.805882 |
| ITM5_personal_victimization | Binary logistic regression | Personal victimization vs never | 603 | age_group=46+ | 0.451556 | 1.570754 | 0.846579 | 2.914397 | 0.152189 |
| ITM5_personal_victimization | Binary logistic regression | Personal victimization vs never | 603 | gender=Male | 1.288306 | 3.626638 | 2.309 | 5.696191 | 2.24E-08 |
| ITM5_personal_victimization | Binary logistic regression | Personal victimization vs never | 603 | residence=Urban | -0.39866 | 0.671217 | 0.429533 | 1.048888 | 0.08005 |
| ITM5_personal_victimization | Binary logistic regression | Personal victimization vs never | 603 | org_type=Public | 0.303305 | 1.354328 | 0.808289 | 2.269244 | 0.249422 |
| ITM5_personal_victimization | Binary logistic regression | Personal victimization vs never | 603 | org_size=10-49 | -0.12711 | 0.880635 | 0.540563 | 1.43465 | 0.60971 |
| ITM5_personal_victimization | Binary logistic regression | Personal victimization vs never | 603 | org_size=50-249 | -1.30241 | 0.271875 | 0.128112 | 0.576967 | 0.000693 |
| ITM5_personal_victimization | Binary logistic regression | Personal victimization vs never | 603 | org_size=250+ | -0.84898 | 0.42785 | 0.201783 | 0.907192 | 0.026831 |
| ITM5_indirect_victimization | Binary logistic regression | Indirect victimization vs never | 862 | const | -0.5116 | 0.599535 | 0.42706 | 0.841667 | 0.003118 |
| ITM5_indirect_victimization | Binary logistic regression | Indirect victimization vs never | 862 | country=Ukraine | 0.496349 | 1.642712 | 1.146534 | 2.353619 | 0.006825 |
| ITM5_indirect_victimization | Binary logistic regression | Indirect victimization vs never | 862 | country=Republic of Moldova | 0.922023 | 2.514372 | 1.756531 | 3.599176 | 4.7E-07 |
| ITM5_indirect_victimization | Binary logistic regression | Indirect victimization vs never | 862 | age_group=26-45 | -0.07365 | 0.928998 | 0.645763 | 1.336461 | 0.691427 |
| ITM5_indirect_victimization | Binary logistic regression | Indirect victimization vs never | 862 | age_group=46+ | 0.605044 | 1.831332 | 1.221855 | 2.744825 | 0.003385 |
| ITM5_indirect_victimization | Binary logistic regression | Indirect victimization vs never | 862 | gender=Male | 0.176247 | 1.192733 | 0.824093 | 1.726274 | 0.350135 |
| ITM5_indirect_victimization | Binary logistic regression | Indirect victimization vs never | 862 | residence=Urban | -0.43562 | 0.646861 | 0.48213 | 0.867878 | 0.003674 |
| ITM5_indirect_victimization | Binary logistic regression | Indirect victimization vs never | 862 | org_type=Public | 0.284378 | 1.328935 | 0.962737 | 1.834424 | 0.083797 |
| ITM5_indirect_victimization | Binary logistic regression | Indirect victimization vs never | 862 | org_size=10-49 | -0.01924 | 0.980939 | 0.695822 | 1.382884 | 0.912541 |
| ITM5_indirect_victimization | Binary logistic regression | Indirect victimization vs never | 862 | org_size=50-249 | -0.24746 | 0.780779 | 0.515272 | 1.183095 | 0.243194 |
| ITM5_indirect_victimization | Binary logistic regression | Indirect victimization vs never | 862 | org_size=250+ | -0.67237 | 0.510498 | 0.305283 | 0.853662 | 0.010374 |
| ITM6_external_vs_manager | Binary logistic regression | External/formal reporting vs manager | 944 | const | -0.6226 | 0.536549 | 0.383246 | 0.751176 | 0.000287 |
| ITM6_external_vs_manager | Binary logistic regression | External/formal reporting vs manager | 944 | country=Ukraine | 0.350825 | 1.420238 | 0.98051 | 2.057171 | 0.063476 |
| ITM6_external_vs_manager | Binary logistic regression | External/formal reporting vs manager | 944 | country=Republic of Moldova | -0.14065 | 0.868793 | 0.604558 | 1.248519 | 0.447111 |
| ITM6_external_vs_manager | Binary logistic regression | External/formal reporting vs manager | 944 | age_group=26-45 | -0.90433 | 0.404811 | 0.276309 | 0.593075 | 3.46E-06 |
| ITM6_external_vs_manager | Binary logistic regression | External/formal reporting vs manager | 944 | age_group=46+ | -0.92755 | 0.395521 | 0.257541 | 0.607427 | 2.26E-05 |
| ITM6_external_vs_manager | Binary logistic regression | External/formal reporting vs manager | 944 | gender=Male | 0.805366 | 2.237516 | 1.591867 | 3.145037 | 3.55E-06 |
| ITM6_external_vs_manager | Binary logistic regression | External/formal reporting vs manager | 944 | residence=Urban | 0.06708 | 1.069381 | 0.79224 | 1.443473 | 0.661175 |
| ITM6_external_vs_manager | Binary logistic regression | External/formal reporting vs manager | 944 | org_type=Public | 0.286917 | 1.332313 | 0.953187 | 1.862235 | 0.093085 |
| ITM6_external_vs_manager | Binary logistic regression | External/formal reporting vs manager | 944 | org_size=10-49 | 0.341423 | 1.406949 | 1.005217 | 1.96923 | 0.046558 |
| ITM6_external_vs_manager | Binary logistic regression | External/formal reporting vs manager | 944 | org_size=50-249 | -0.4199 | 0.657111 | 0.419492 | 1.029328 | 0.066694 |
| ITM6_external_vs_manager | Binary logistic regression | External/formal reporting vs manager | 944 | org_size=250+ | -0.88034 | 0.414642 | 0.235483 | 0.730107 | 0.002291 |
| ITM6_no_action_vs_manager | Binary logistic regression | No action vs manager | 668 | const | -3.68743 | 0.025036 | 0.010001 | 0.062675 | 3.38E-15 |
| ITM6_no_action_vs_manager | Binary logistic regression | No action vs manager | 668 | country=Ukraine | 1.340915 | 3.822541 | 1.650205 | 8.85455 | 0.001756 |
| ITM6_no_action_vs_manager | Binary logistic regression | No action vs manager | 668 | country=Republic of Moldova | -1.16912 | 0.310639 | 0.081505 | 1.18393 | 0.08678 |
| ITM6_no_action_vs_manager | Binary logistic regression | No action vs manager | 668 | age_group=26-45 | -1.16417 | 0.31218 | 0.118985 | 0.819067 | 0.018005 |
| ITM6_no_action_vs_manager | Binary logistic regression | No action vs manager | 668 | age_group=46+ | -0.45997 | 0.631305 | 0.270612 | 1.472757 | 0.287222 |
| ITM6_no_action_vs_manager | Binary logistic regression | No action vs manager | 668 | gender=Male | 0.698059 | 2.009847 | 0.895122 | 4.512777 | 0.090743 |
| ITM6_no_action_vs_manager | Binary logistic regression | No action vs manager | 668 | residence=Urban | 1.003513 | 2.727847 | 1.325028 | 5.615842 | 0.006452 |
| ITM6_no_action_vs_manager | Binary logistic regression | No action vs manager | 668 | org_type=Public | 1.991992 | 7.330121 | 3.375475 | 15.91796 | 4.78E-07 |
| ITM6_no_action_vs_manager | Binary logistic regression | No action vs manager | 668 | org_size=10-49 | -0.67697 | 0.508155 | 0.216985 | 1.190046 | 0.118944 |
| ITM6_no_action_vs_manager | Binary logistic regression | No action vs manager | 668 | org_size=50-249 | -1.63633 | 0.194694 | 0.056106 | 0.675608 | 0.009946 |
| ITM6_no_action_vs_manager | Binary logistic regression | No action vs manager | 668 | org_size=250+ | -0.64848 | 0.522839 | 0.171332 | 1.595507 | 0.25461 |
| ITM7_non_refusal | Binary logistic regression | Conditional acceptance/ tolerance vs no | 987 | const | -2.74419 | 0.064301 | 0.036974 | 0.111824 | 2.48E-22 |
| ITM7_non_refusal | Binary logistic regression | Conditional acceptance/ tolerance vs no | 987 | country=Ukraine | 0.877796 | 2.405591 | 1.379095 | 4.196136 | 0.001986 |
| ITM7_non_refusal | Binary logistic regression | Conditional acceptance/ tolerance vs no | 987 | country=Republic of Moldova | 0.798619 | 2.222468 | 1.291819 | 3.823574 | 0.003915 |
| ITM7_non_refusal | Binary logistic regression | Conditional acceptance/ tolerance vs no | 987 | age_group=26-45 | -0.34323 | 0.709478 | 0.412658 | 1.219797 | 0.21447 |
| ITM7_non_refusal | Binary logistic regression | Conditional acceptance/ tolerance vs no | 987 | age_group=46+ | -0.7764 | 0.460057 | 0.24321 | 0.870247 | 0.016973 |
| ITM7_non_refusal | Binary logistic regression | Conditional acceptance/ tolerance vs no | 987 | gender=Male | 1.186789 | 3.276542 | 2.099751 | 5.112858 | 1.72E-07 |
| ITM7_non_refusal | Binary logistic regression | Conditional acceptance/ tolerance vs no | 987 | residence=Urban | -0.49849 | 0.607448 | 0.38791 | 0.951236 | 0.029373 |
| ITM7_non_refusal | Binary logistic regression | Conditional acceptance/ tolerance vs no | 987 | org_type=Public | -0.0762 | 0.92663 | 0.567361 | 1.5134 | 0.760785 |
| ITM7_non_refusal | Binary logistic regression | Conditional acceptance/ tolerance vs no | 987 | org_size=10-49 | 0.505842 | 1.658381 | 1.011933 | 2.717796 | 0.044747 |
| ITM7_non_refusal | Binary logistic regression | Conditional acceptance/ tolerance vs no | 987 | org_size=50-249 | 0.100911 | 1.106179 | 0.562648 | 2.174774 | 0.769849 |
| ITM7_non_refusal | Binary logistic regression | Conditional acceptance/ tolerance vs no | 987 | org_size=250+ | -0.35386 | 0.701972 | 0.307389 | 1.603067 | 0.400976 |
| ITM8_colleague_involvement_yes | Binary logistic regression | Colleague involvement yes vs no/don't know | 987 | const | -3.01597 | 0.048998 | 0.027456 | 0.087445 | 1.87E-24 |
| ITM8_colleague_involvement_yes | Binary logistic regression | Colleague involvement yes vs no/don't know | 987 | country=Ukraine | 0.748994 | 2.114872 | 1.184251 | 3.776804 | 0.011356 |
| ITM8_colleague_involvement_yes | Binary logistic regression | Colleague involvement yes vs no/don't know | 987 | country=Republic of Moldova | 1.036688 | 2.819861 | 1.63164 | 4.873389 | 0.000204 |
| ITM8_colleague_involvement_yes | Binary logistic regression | Colleague involvement yes vs no/don't know | 987 | age_group=26-45 | -0.06904 | 0.933289 | 0.536104 | 1.624737 | 0.807165 |
| ITM8_colleague_involvement_yes | Binary logistic regression | Colleague involvement yes vs no/don't know | 987 | age_group=46+ | 0.033371 | 1.033934 | 0.578455 | 1.848059 | 0.910331 |
| ITM8_colleague_involvement_yes | Binary logistic regression | Colleague involvement yes vs no/don't know | 987 | gender=Male | 0.796209 | 2.217119 | 1.415607 | 3.472446 | 0.000505 |
| ITM8_colleague_involvement_yes | Binary logistic regression | Colleague involvement yes vs no/don't know | 987 | residence=Urban | -0.05082 | 0.950449 | 0.612038 | 1.475978 | 0.820964 |
| ITM8_colleague_involvement_yes | Binary logistic regression | Colleague involvement yes vs no/don't know | 987 | org_type=Public | 0.438811 | 1.550862 | 0.968867 | 2.482459 | 0.06752 |
| ITM8_colleague_involvement_yes | Binary logistic regression | Colleague involvement yes vs no/don't know | 987 | org_size=10-49 | 0.129061 | 1.13776 | 0.69137 | 1.872366 | 0.611595 |
| ITM8_colleague_involvement_yes | Binary logistic regression | Colleague involvement yes vs no/don't know | 987 | org_size=50-249 | -1.03211 | 0.356256 | 0.155783 | 0.814713 | 0.014465 |
| ITM8_colleague_involvement_yes | Binary logistic regression | Colleague involvement yes vs no/don't know | 987 | org_size=250+ | 0.330926 | 1.392256 | 0.711423 | 2.724649 | 0.334032 |

Notes: Holm-adjusted p-values for the adjusted outcome analyzes are reported in Table 7 and were calculated only across the 16 prespecified national-subsample contrasts. In this supplementary table, covariate coefficients are presented for adjustment transparency and are not interpreted as primary hypothesis tests.

* Note: ORs are from ordinal logistic models for ITM_4 and ITM_11 and binary logistic models for prespecified contrasts from ITM_5, ITM_6, ITM_7, and ITM_8. Romania is the reference category for national subsample membership. Models adjust for age group, gender, residence environment, organization type, and organization size. Reference categories were Romania for national subsample membership, age under 25 years, female gender, rural residence, private-sector organization, and organizations with fewer than 10 employees. For ordinal outcomes, OR>1 indicates higher odds of being in a higher response category; for binary outcomes, OR>1 indicates higher odds of the event listed in the Level_or_event column.

**Supplementary Table 3.** National subsamples distribution of Likert responses for ITM 4 (potentially fraudulent/unethical activity proposals) and ITM 11 (gift acceptance)

| Country | R1 | R2 | R3 | R4 | R5 | High-frequency category |
| --- | --- | --- | --- | --- | --- | --- |
| ITM 4 – “Have you ever been asked to participate in fraudulent conduct?” | | | | | | |
| Romania | 71% | 10% | 9.8% | 4.9% | 3.4% | 18.2% |
| Ukraine | 52% | 19% | 17.7% | 5.4% | 5.4% | 28.6% |
| Republic of Moldova | 68% | 14% | 12.4% | 2.8% | 2.0% | 17.2% |
| ITM 11 – “gifts/hospitality acceptance” | | | | | | |
| Romania | 66% | 13.5% | 13.0% | 3.7% | 3.4% | 7.1% |
| Ukraine | 49.7% | 21.4% | 16.0% | 7.4% | 5.4% | 12.9% |
| Republic of Moldova | 60% | 15.6% | 14.4% | 7.2% | 2.8% | 10.0% |

Notes: For ITM_4, the final column reports categories 3-5; for ITM_11, it reports categories 4-5 (“often/very often”).

**Supplementary Table 4**. Cluster profile by key MCA input items

| Item | Cluster 1: dominant / defining response | Cluster 2: dominant / over-represented response | Contrast measure | Interpretation |
| --- | --- | --- | --- | --- |
| ITM_2 financial distortions | No: 68.5% | Yes: 78.0% | Lift C2 vs overall=3.71 | Cluster 2 is strongly defined by reported exposure to financial distortions. |
| ITM_5 victimization | Never: 55.9% | Yes: 62.4% | Lift C2 vs overall=4.99 | Cluster 2 is strongly defined by personal victimization. |
| ITM_6 reporting channel | Manager: 65.4% | State/anti-fraud authority: 46.1% | Lift C2 vs overall=1.42 | Cluster 2 shows relatively greater orientation toward external/formal reporting. |
| ITM_7 conditional acceptance/refusal under superior pressure | No: 96.3% | Yes, if I also benefit: 44.7%; Depends: 12.8% | Lift C2 vs overall=6.82 for “Yes”; 2.74 for “Depends” | Cluster 2 is defined by a higher share of non-refusal responses to superior-requested fraud. |
| ITM_8 colleague practices | No: 67.1% | Yes: 61.7% | Lift C2 vs overall=5.97 | Cluster 2 is strongly defined by perceived colleague involvement. |
| ITM_10 fraud-risk management | Risk assessment/control system: 65.5% | Risk assessment/control system: 48.2%; reporting events: 41.1% | Lift C2 vs overall=1.42 for “reporting events” | Cluster 2 retains control-oriented responses but shows stronger emphasis on reporting suspicious events. |

**Supplementary Table 5**. Proportional-odds diagnostics for ordinal outcome models

| Outcome | National-subsample contrast | OR >1 vs ≤1 | OR >2 vs ≤2 | OR >3 vs ≤3 | OR >4 vs ≤4 | Threshold-specific OR range | Evidence of material threshold variation | Interpretation |
| --- | --- | --- | --- | --- | --- | --- | --- | --- |
| ITM_4 | Ukraine vs Romania | 2.44 | 2.11 | 1.52 | 1.24 | 1.24–2.44 | No strong material variation | Positive contrast across all cumulative thresholds; effect stronger at lower thresholds. |
| ITM_4 | Republic of Moldova vs Romania | 1.16 | 0.93 | 0.51 | 0.56 | 0.51–1.16 | Limited/unstable contrast | No consistent positive adjusted contrast; interpreted cautiously. |
| ITM_11 | Ukraine vs Romania | 1.85 | 1.47 | 1.60 | 2.33 | 1.47–2.33 | No strong material variation | Positive contrast across all cumulative thresholds. |
| ITM_11 | Republic of Moldova vs Romania | 1.30 | 1.31 | 1.62 | 1.15 | 1.15–1.62 | No strong material variation | Modest positive contrast across thresholds, but not retained as a main adjusted finding. |

Notes. Values are adjusted odds ratios from threshold-specific cumulative binary logistic regression models fitted at each cut-point using the same covariates as the main adjusted ordinal models: national subsample, age group, gender, residence environment, organization type, and organization size. The analytical sample for these diagnoses was N=987, after excluding non-informative gender and organization-type responses. OR>1 indicates higher adjusted odds of being above the respective cumulative threshold. The diagnostics focus on national-subsample contrasts because these were the inferential quantities of interest. Some threshold variation was observed for organization-size covariates in the ITM_11 model; therefore, multinomial sensitivity models were also estimated.
